# Heterogeneity and nonlinearity in consumers’ preferences: An application to the olive oil shopping behavior in Chile

**DOI:** 10.1371/journal.pone.0184585

**Published:** 2017-09-11

**Authors:** Rodrigo Alejandro Romo-Muñoz, Juan Hernán Cabas-Monje, Héctor Manuel Garrido-Henrríquez, José María Gil

**Affiliations:** 1 Agribusiness Research Group, Business Management Department, Universidad del Bío-Bío, Chillán, Chile; 2 Center for Agro-food Economy and Development (CREDA-UPC-IRTA), Parc Mediterrani de la Tecnologia, Edifici ESAB, Castelldefels, Barcelona, Spain; Middlesex University, UNITED KINGDOM

## Abstract

In relatively unknown products, consumers use prices as a quality reference. Under such circumstances, the utility function can be non-negative for a specific price range and generate an inverted U-shaped function. The extra virgin olive oil market in Chile is a good example. Although domestic production and consumption have increased significantly in the last few years, consumer knowledge of this product is still limited. The objective of this study was to analyze Chilean consumer preferences and willingness to pay for extra virgin olive oil attributes. Consumers were segmented taking into account purchasing frequency. A Random Parameter Logit model was estimated for preference heterogeneity. Results indicate that the utility function is nonlinear allowing us to differentiate between two regimes. In the first regime, olive oil behaves as a conspicuous good, that is, higher utility is assigned to higher prices and consumers prefer foreign products in smaller containers. Under the second regime, Chilean olive oil in larger containers is preferred.

## Introduction

Chile only represents 0.2% of the world olive tree cultivated area and 0.6% of world olive oil production. However, establishment of this crop has been remarkable and with exponential growth rates (from 192 tons in 1997 to 21 600 tons in 2012). This significant increase has been favored by foreign investment from producer countries that have established themselves in Chile, which have been attracted by suitable climatic conditions and relatively low land prices compared with European countries.

Consumption (per capita) has also exhibited significant growth, going from 77 mL in 1997 to 824 mL in 2011; these figures are still far from those found in the main producer countries such as Spain, Italy, and Greece. Extra virgin olive oil is a relatively new product in Chile and it is at the introduction stage. Olive oil was initially imported from Spain or Italy at a price that was much higher than sunflower oil (the most consumed oil in the country). Increased production has allowed the Chilean consumer to obtain a high-quality product at a competitive price compared with the imported product. Most producers have targeted the domestic market although the Chilean consumer is somewhat ignorant about this product.

The objective of this study was to analyze Chilean consumer preferences for extra virgin olive oil by focusing on price and origin attributes and the possibility of preference heterogeneity. The knowledge generated about attribute valuation in the different markets is relevant for companies wishing to enter these markets or for local producers interested in developing their own new market. Companies can use this information to develop appropriate marketing strategies and minimize the possibility of a product launch failure. This is the first attempt to analyze consumer preferences towards extra virgin olive oil in Chile.

The methodological approach was based on conducting a Choice Experiment (CE) with a representative sample of 221 consumers from the Biobío Region in Chile, which is the second most important region after the Metropolitan Region in terms of economy and population. It is also the region with the highest potential for growth of olive oil production mainly due to its climatic and agronomic conditions [1]. The CE method has been used extensively in the literature. It has been proved to be very useful for collecting information from consumers about their preferences and how they assess each product attribute and its different levels, especially when dealing with complex goods such as food products [2]. Its popularity has increased over the last few years because: i) it is easy to implement; ii) it mimics real shopping scenarios by also including a no-choice alternative, which reduces bias that might exist when applying it [3]; and iii) preference heterogeneity can be easily considered [4]. Despite these interesting advantages, the CE also presents certain limitations, among which the most relevant is that in many applications the CE does not consider person’s budget constraint, which can overestimate participants’ WTP [5]. In any case, [6] has suggested some ex ante survey design to reduce hypothetical bias in CE.

Empirical applications of CE cover a wide range of scientific fields: Marketing [7]; Environmental Economics [8]; Transport [9]; Health Economics [10]; or Energy [11]. Applications in food markets have been concentrated mainly in meat [12] [13] [14] [15]; wine [16] [17] [18] [19]; milk and dairy products [20] [21]; or fruit [22] [23].

In most studies, the price variable has been introduced as a linear function and results between utility and price are inversely related as suggested by economic theory. However, in some cases, such as for prestigious goods or goods that are relatively unknown by consumers, this negative relationship does not need to be fulfilled [24], at least for the whole price range. Under these circumstances, price can be perceived as a quality indicator and a positive relationship can exist between price and utility for low and medium price levels [25] [26]. Nevertheless, taking into account the budget restriction, there will always be a threshold price at which the relationship will be negative again. The possibility of a nonlinear relationship between price and utility is the second contribution of the present study.

The empirical applications of CE to the olive oil sector are limited. Our search has identified 78 studies that focus on olive oil consumer preferences, of which 73% have been conducted in Mediterranean countries, 27% in North European countries, and only 10% in the rest of the world [27]. Most of the studies carried out in Mediterranean countries are focused on the largest producing countries [4] [28] [29] [30] [31] [32] [33] [34]. Only three studies have been conducted in developed and non-producer countries with high purchasing power (high consumption potential), such as the United Kingdom [35], Japan [36], and Canada [3].

Results from previous literature depend on two main issues: 1) the regulations currently in force for olive oil production in each country; and 2) the consumer’s experience in using olive oil. The legal regulation determines the different types of intrinsic (e.g. acidity, olive variety) and extrinsic (e.g. brand, denomination of origin) olive oil attributes, which are reported by the producers on the product label. This, together with consumer experience in using the product, determines the valuation and importance given to it at the time of purchase. In the case of studies conducted in traditional countries, the most sophisticated attributes are those valued by consumers. Some attributes such as the protected designation of origin (PDO), declaration of health properties, production quality certifications, and sensory attributes are the most valued in these markets [27] [37]. This is evident in some countries such as Greece [31] [34] and Italy [24] [32], and Spain [30]. In studies carried out in non-producer markets, the most relevant attributes are price and oil color. These results are given in the context of less strict regulation and with inexperienced consumers in the use of the product.

The rest of the study is as follows. Section 2 describes the materials and methods. Section 3 outlines the empirical application. Section 4 discusses the main results obtained in this study. Finally, section 5 presents some concluding remarks.

## Materials and methods

The Choice Experiment (CE) is based on the fundamental principles of economic theory because it is consistent with Lancaster’s microeconomic approach [38] and the random utility theory (RUT) [39]. Choice experiments assume that individuals are rational and make their decisions to maximize their utility based on their budget restriction. Individuals select an alternative (from various options) in accordance with a function expressed as

$$U_{in}=V_{in}(Z_{i},S_{n})+\varepsilon_{in}$$ (1)

Where *U*_*in*_ is the utility provided by alternative *i* for individual *n*. This function has a systematic utility component *(V*_*in*_*)* and a random component *(ε*_*in*_*)*. *Z*_*i*_ is the attribute of each alternative and *S*_*n*_ is the vector of the socioeconomic characteristics of the survey respondents. Based on this function, the CE model proposes that an individual *n* will always choose alternative *i* if it provides higher utility than alternative *j*. In terms of probability, choosing *i* can be expressed as

$$P_{in}=Prob\left[U_{in}>U_{jn}\right]=Prob\left[V_{in}+\varepsilon_{in}>V_{jn}+\varepsilon_{jn}\right]\forall i\neq j$$ (2)

This expression is equivalent to

$$Prob\left[\varepsilon_{jn}-\varepsilon_{in}<V_{in}-V_{jn}\right]\forall i\neq j$$ (3)

[39] proposed an econometric framework to estimate discrete choice models based on random utility models in which consumers were assumed to be homogeneous; this implies that all coefficients for all attributes considered in the utility function are constant across the sample. To overcome this restrictive assumption, several alternatives have been proposed in the literature: the Random Parameter Logit Model (RPL); the Latent Class Model (LCM); or the Independent Availability Logit (IAL) model, among others. In this paper, we have specified the use of the RPL to be consistent with previous literature on consumer’s preferences for extra virgin olive oil. The RPL model is a generalization of the Multinomial Logit Model (MNL) that allows accounting for preference heterogeneity among individuals. In this case, expression (1) becomes:

$$U_{in}=V_{in}(Z_{i},S_{n})+\varepsilon_{in}=\beta \prime x_{in}+\varepsilon_{in}$$ (4)

The RPL model is based on the assumption that the observed portion of the utility depends on the vector of parameters *β* that are random and distributed across individuals according to a statistical distribution reflecting preference heterogeneity. The *x*_*in*_ is a vector of attributes corresponding to alternative *i*. The unconditional probability to choose alternative *i* at time *t*, *P*_*in*_ (considering that consumers adopt sequential decisions), is expressed as:

$$P_{in}(\beta )=\int \left[\frac{e^{\beta \prime x_{in}}}{\sum_{j}e^{\beta \prime x_{jn}}}\right]f\left(\beta |\theta \right)d$$ (5)

The distinctive feature of this function is that the parameter distribution is characterized by the mean and variance (represented by *θ*) since it is not possible to directly obtain the parameters. It can be deduced from the above formula that the probabilities of each choice are weighted by a fixed probability density function set at the discretion of the researcher (usually uniform, triangular, normal, or lognormal). Parameters are random variables and all possible values must be integrated to obtain the unconditional probability of *P*_*in*_. Given that the above formula has no analytical solution, it must be estimated by simulation methods.

Based on the assumption that price is a linear utility function, willingness to pay (WTP) for each attribute has traditionally been calculated by dividing the attribute coefficient of the selected attribute *(β*_*j*_*)* by the coefficient associated with price *(β*_*price*_*)*, which is expressed as $WTP=-\frac{\beta_{j}}{\beta_{price}}$ [40] [41] [42] [43]. However, as previously mentioned in the introduction, the utility function is nonlinear in some cases; this occurs mainly in cases where the product is relatively new for consumers. The utility function can therefore be represented by an inverted U-shaped parabola. For a certain price range, utility increases with price (which is used by consumers as a proxy for quality) up to a price threshold at which the utility function changes to a negative slope. The Price Threshold *(P*_*threshold*_*)* can be calculated by deriving the nonlinear utility function with respect to price [21]:

$$U=\beta_{p}P+\beta_{p^{2}}p^{2}+\sum \beta X$$ (6)

$$\frac{\partial U}{\partial p}=\beta_{p}+2\beta_{p^{2}}p$$ (7)

where *X* represents the product attributes except price, *β*_*p*_ is the price coefficient in the utility function, and $\beta_{p^{2}}$ is the coefficient of price squared. If *β*_*p*_ > *0* and $\beta_{p^{2}}<0$, the indirect utility function becomes parabolic. The *P*_*threshold*_ is obtained by equalizing expression (7) to zero:

$$P_{threshold}=-\frac{\beta_{p}}{2\beta_{p^{2}}}$$ (8)

The indirect utility function is maximized at the *P*_*threshold*_. The utility function has a positive slope in relation to price when price is lower than *P*_*threshold*_; however, it is positive when price is higher than *P*_*threshold*_. In this model, WTP for each attribute was calculated for both regimes of the utility function.

## Empirical application

### The sample

To achieve the study objectives, a survey was specifically designed and applied to a representative sample of the population in the Biobío Region, which is the second most populated region of Chile (the survey was approved by the Ethics Committee at the University of Bío-Bío and did not contain any identification information) (S1 File). Survey respondents were responsible for household purchases and declared having bought extra virgin olive oil at least once during the last three months. A total of 221 valid responses was obtained. The sample was stratified by gender, age, and residential area. The information needed to stratify the sample was obtained from the Chilean Instituto Nacional de Estadísticas [44]. Respondents were recruited outside the main mall at the center of the town as well as outside the five more important supermarkets. A letter of consent was signed by each participant before starting the survey and after checking their sociodemographic characteristics to guarantee representativeness (S2 File).

The survey included three sections. The first section characterized consumer olive oil shopping behavior as well as consumption habits. The second section addressed the CE from which survey respondents had to select the alternative in each choice set that was closest to their real behavior. In order to reduce the potential hypothetical bias, a specific cheap talk was introduced to remind the respondents that choices have to be made taking into account what they really buy and not what they would prefer buying. The last section included socio-demographic information about the survey respondents (lifestyle, marital status, education, type of occupation, and income). The field work took place between January and March 2012.

### Choice experiment design

The final set of attributes and levels in the CE was based on both the literature review of similar studies carried out in non-traditional producer countries and qualitative research based on the observation of olive oil products available in different supermarkets throughout the Biobío Region. Most of the attributes used in previous studies are only applicable in traditional markets. Attributes such as color, texture, taste, or odor cannot be considered in Chile because consumers are not sufficiently familiarized with this product. Furthermore, both producers and retailers only include the minimum label information required by the authorities. In studies conducted in emergent markets, attributes and attribute levels have been defined by taking into account the specific location where the study has been carried out. In this framework, two focus groups were also conducted; each group included eight extra virgin olive oil consumers. The final set of attributes and attribute levels are shown in Table 1.

**Table 1:** Attributes and attribute levels for each extra virgin olive oil.

Levels Attributes	1	2	3	4	5	6
**Country of origin**	Chile	Spain	Italy			
**Type of container**	Glass	Plastic	Metal			
**Container size**	250 mL	500 mL	1000 mL			
**Price (CLP)**	$1900	$2500	$3000	$4100	$5100	$6100

CLP: Chilean peso (€1 = CLP $650 on average during the field work)

**Fig 1:**
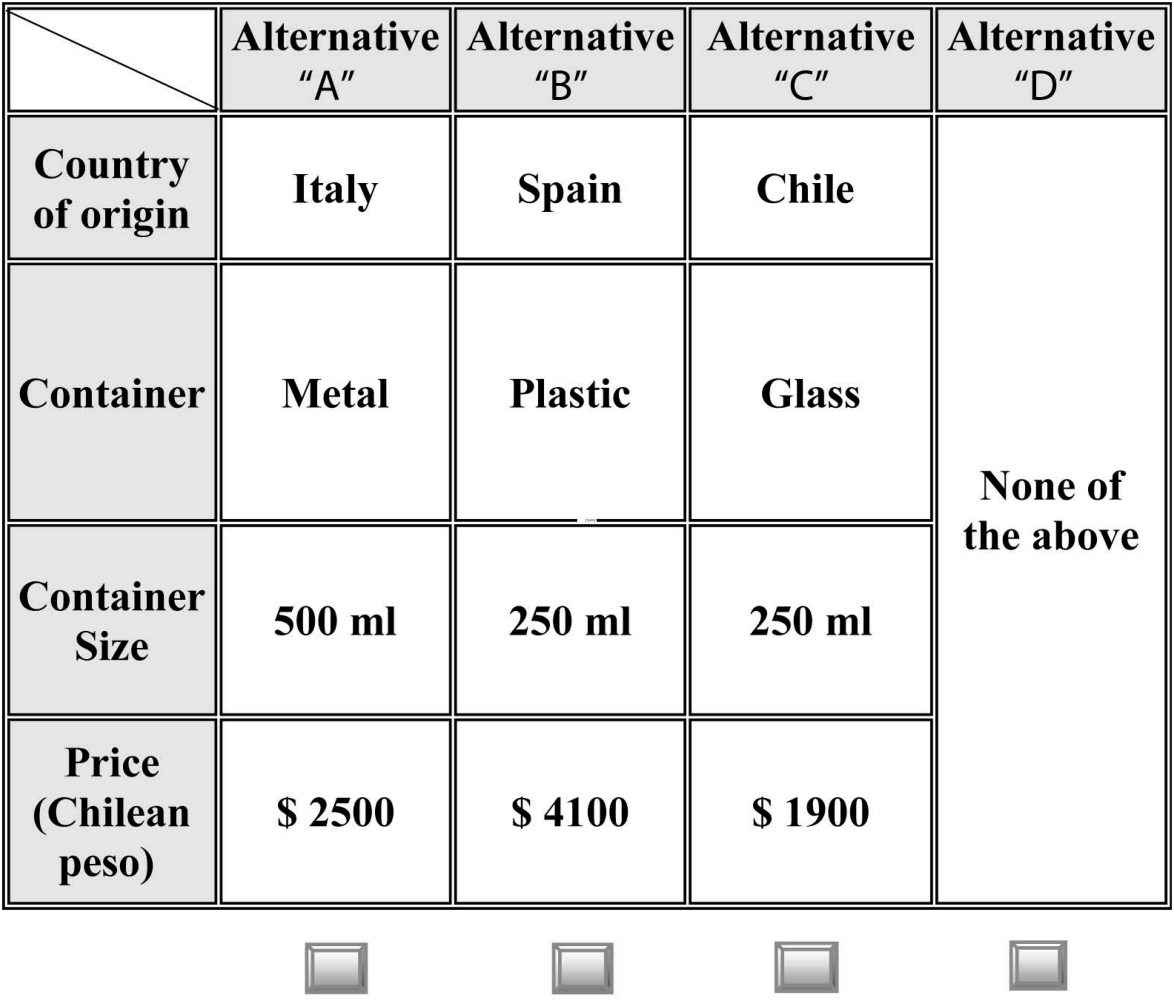
Example of choice card.

Combining attributes and attribute levels resulted in a full factorial design of 162 hypothetical product combinations or choice sets. Facing respondents with 162 choice sets could place a high level of cognitive burden on them. To reduce the number of combinations that participants have to evaluate, we followed [45] and we generated an orthogonal fractional factorial design in which a random block was also introduced [46], resulting in 27 hypothetical combinations. These 27 combinations were considered as the first option in each choice set. Since participants were provided with choice sets of 3 options each (plus a no-choice option), the other two options were obtained using the generators (1, 1, 2, 3) and (2, 2, 1, 4) [45]. This resulted in a 100% efficient main-effects design. The 27 hypothetical product combinations were equally divided into three blocks of nine cards (choice sets). Each block was randomly assigned to one third of the respondents. Fig 1 shows one of the choice sets offered to respondents.

## Results

Given that extra virgin olive oil is a relatively new product in Chile, the sample was divided into two main segments based on their expertise in olive oil. Expertise was estimated by consumption frequency. Thus, the two segments were self-identified in the survey as regular and occasional extra virgin olive oil consumers. Two types of models were estimated for each market segment. The first considered a linear utility function while the second assumed a quadratic function with respect to price.

Parameter estimates from both models are shown in Table 2 (S3 File). It was assumed in both models that parameters associated with attribute origin, container size, and type of container were normally distributed. The normal distribution is suitable for the purpose of this study because no a priori assumptions have been made about their sign. Parameters associated to price have been specified as being fixed.

**Table 2:** Random parameter logit estimates for the linear and nonlinear models^a^^,^^b^^,^^c^^,^^d^.

	Estimated Coefficients		Standard Deviation	
Variable	Linear	Nonlinear	Linear	Nonlinear
No-option	27.6224	33.0725	-	-
	[0.02]	[0.01]	-	-
Spain	1.2927***	0.573***	0.3395	-0.459
	[7.00]	[2.90]	[1.13]	[-0.96]
Chile	1.8141***	1.359***	1.4303 ***	1.011***
	[7.05]	[5.66]	[5.40]	[3.47]
Metal	0.1970	-0.473	2.1362***	2.220***
	[0.72]	[-1.61]	[7.57]	[7.86]
Glass	2.9498***	2.715***	1.7681***	2.008***
	[9.67]	[8.15]	[7.57]	[7.87]
500 mL	0.8530***	0.613***	0.3115	0.538***
	[5.86]	[4.11]	[0.98]	[2.33]
1000 mL	0.9564***	0.655	1.7260***	1.605***
	[4.20]	[2.97]	[7.24]	[5.56]
Price	-0.004	1.551***	-	-
	[-0.13]	[7.96]	-	-
Price^2^	-	-0.219***	-	-
	-	[-8.30]	-	-
Spain_regular_	-0.1749	-0.353	-1.2819***	0.763
	[-0.43]	[-0.92]	[3.20]	[1.11]
Chile_regular_	0.986*	0.307	0.4857	1.500**
	[1.94]	[0.65]	[1.00]	[2.41]
Metal_regular_	0.2896	0.469	0.4954	-1.131
	[0.65]	[0.82]	[-0.17]	[-1.33]
Glass_regular_	0.6769	0.778	2.027***	1.369**
	[1.32]	[1.4]	[-4.51]	[2.49]
500 mL_regular_	0.3084	0.281	0.5748*	-0.373
	[1.11]	[0.96]	[1.71]	[-0.96]
1000 mL_regular_	0.9908**	0.727	-0.5294 **	2.142***
	[2.30]	[1.54]	[-1.92]	[3.27]
Price_regular_	-0.1137**	0.293	-	
	[-2.01]	[0.78]	-	
Price^2^_regular_	-	-0.053	-	
	-	[-1.05]	-	
Log likelihood [*χ*^2^] -1120.413 [214.93] -1050.03 [216.42]				
AIC/BIC	2294.83 / 2473.75 2158.06 / 2350.24			
N	5580	5580		

^**a**^ *p < 0.1; **p < 0.05; ***p < 0.01.

^**b**^ t ratios in square brackets.

^**c**^ Each model consisted of 1000 random samples, the positive value must be interpreted.

^**d**^ Base categories are Italy, plastic and 250 mL for each attribute level.

**Fig 2:**
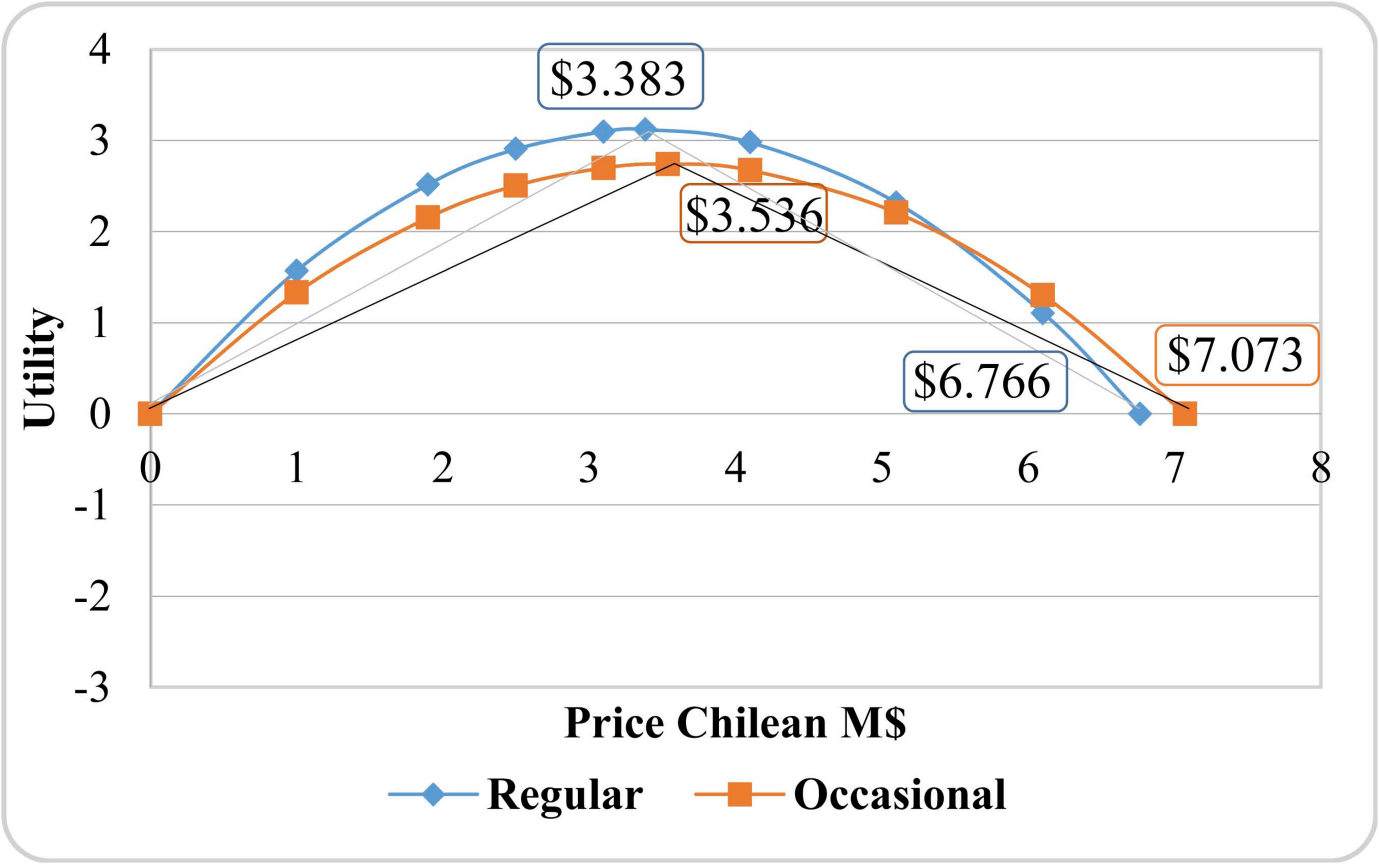
Relationship between utility and extra virgin olive oil price in Chile.

Results indicate (Table 2) that in both models the no-option coefficient is not significant, indicating that frequently respondents have chosen the option “none of them”. This result is consistent in an emerging market in which consumers are still not very acknowledgeable about extra virgin olive oil characteristics. In the linear model, the price parameter is negative but nonsignificant, making it impossible to calculate the Chilean consumer’s WTP for extra virgin olive oil attributes based on the expression shown in section 2. In the nonlinear model, price coefficients are significant at the 1% significance level. The parameter associated to price is positive while it is negative for price^2^ (squared) indicating that the utility function is represented by an inverted U-shaped parabola. Consumer utility increases when price increases to a certain threshold (CLP$3383 and CLP$3536 for regular and occasional consumers, respectively, according to Eq 8) (Fig 2).

It is also interesting to note that in all cases, except for the Spanish origin, standard deviation parameters are positive, thus indicating preference heterogeneity in Chilean consumers in relation to extra virgin olive oil and the adequacy of estimating the RPL model. Moreover, no significant differences were found between occasional and regular consumers (all interaction parameters in Table 2 are nonsignificant in the nonlinear model). Olive oil is still a relatively unknown product in Chile although some segments show higher frequency and consumption preferences for product attributes do not differ.

Attribute level parameters show that Chilean extra virgin olive oil consumers prefer domestic olive oil over their Italian and Spanish counterparts. As for containers, glass and metal are preferred over plastic (with no significant preference between these two materials) as well as larger container size (the 1L container is preferred); this is consistent with Chilean extra virgin olive oil in retail outlets that are normally in 500 mL or 1000 mL containers.

From the estimated parameters displayed in Table 2, we calculated WTP for the different attribute levels. Given that utility is nonlinear with respect to price, we cannot use the traditional expression to calculate WTP values because the slope is not constant. The quadratic function allows us to differentiate between two different regimes as shown in Fig 2. The first goes from zero to the threshold price (CLP$3383 and CLP$3536, for regular and occasional consumers, respectively), exhibiting a positive slope while the second occurs after the threshold price. In this regime, the slope is negative and utility decreases up to zero, and the price is CLP$6766 and CLP$7073 for regular and occasional consumers, respectively. From this point on, utility is negative.

**Fig 3:**
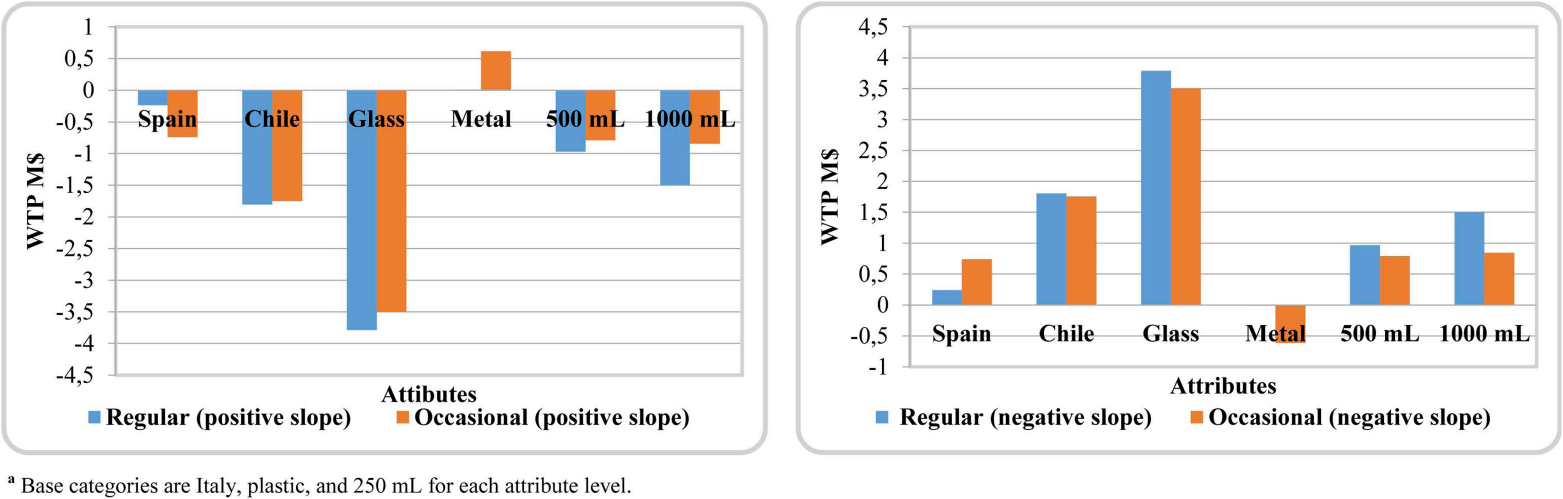
Willingness to pay (WTP) for positive and negative slope utility^a^.

Given this function, WTP was calculated for each regime using the same expression found in the linear model but substituting the price coefficient by the corresponding slope in each regime [26] (Fig 3). For lower prices, results indicate that the utility function of Chilean consumers increases with olive oil from Italy and Spain and packaged in plastic or metal and smaller-sized containers. However, when the price is higher than CLP$3500, their utility function increases with domestically produced olive oil packaged in 1L glass containers. When the price is not very high, this attribute is taken as a proxy for quality and consumers place more value on the foreign product. However, when the price is higher, the Chilean attribute becomes relevant because the price for domestic extra virgin olive oils is lower.

## Conclusion

The present study constitutes the first attempt to calculate the Chilean consumer’s willingness to pay for extra virgin olive oil attributes. Olive oil is still a new product in Chile in spite of significant growth rates of both domestic production and consumption. Chilean consumers are not very knowledgeable about olive oil attributes and may use price as a proxy for quality. Under such circumstances, the utility function cannot be linear with respect to price. In the case of the Chilean olive oil market, results suggest that the utility function can be represented by an inverted U-shaped parabola. Utility increases when price increases up to a threshold and then decreases. The threshold was set at CLP$3383 and CLP$3536 for regular and occasional olive oil consumers, respectively. Results also suggest the existence of preference heterogeneity among Chilean consumers, as most of the estimates of the standard deviations were significant suggesting that a model that accounts for such heterogeneity, such as the RPL, is adequate for the purpose of the present study.

As for the assessment of different product attributes, there is a systematic consumer preference for olive oil that is produced locally and presented in larger glass containers, which is consistent with the existing range of products in Chilean retail stores. Similar results were also found in a non-traditional olive oil market (the British market) by [35]. Nonsignificant differences were found between regular and occasional consumers.

The concavity of the utility function allowed us to differentiate between two regimes. In the first regime, olive oil behaves as a conspicuous good, that is, higher utility is assigned to higher prices and consumers prefer foreign products in smaller containers. Under the second regime, Chilean olive oil in larger containers is preferred.

The role of the price attribute as a proxy for quality has been extensively studied [47] [48] [49] [50]. For specific studies in food, we can mention [51] [52] or [53] among others. Empirical evidence usually suggests that the price/quality inference made by the consumer becomes a strong predictor of consumer behavior when the individual has limited information, but this factor becomes less relevant when more information about the intrinsic aspects of the product is available [50]. In non-traditional markets, such as Germany [54] and England [35], price is also a relevant variable when buying the product.

The preference for Chilean extra virgin olive oil is a result that deserves further attention because of its important policy implications. The government should focus on supporting domestic producers in addition to informing the population about the benefits derived from a more frequent consumption of the product. Efforts in recent years have been directed to promoting exports although the domestic market has important growth opportunities.

## Supporting information

S1 File Questionnaire. (PDF)

S2 File Survey technical information. (DOCX)

S3 File Choice olive oil dataset. (XLSX)
