# Supplementary material for: Heterogeneity and nonlinearity in consumers’ preferences: An application to the olive oil shopping behavior in Chile
Source: PLoS One. 2017 Sep 11;12(9):e0184585. doi: 10.1371/journal.pone.0184585 (PMC5593193; doi:10.1371/journal.pone.0184585)
Supplement: S1 File — (PDF) [file pone.0184585.s001.pdf]

Comité Asesor de Bioética y Bioseguridad
DIRECCIÓN DE INVESTIGACIÓN
Universidad del Bío - Bío

UNIVERSIDAD DEL BÍO-BÍO

Buenos días / Buenas tardes, somos alumn@s de la carrera de Ingeniería Comercial de la Universidad del Bío-Bío y estamos desarrollando un estudio que tiene como objetivo conocer los hábitos de compra y consumo de aceite de oliva. Queremos pedirle si sería tan amable de responder a una breve encuesta, su opinión nos será de gran utilidad. Aseguramos que la información que nos proporcione será utilizada sólo con fines de investigación.

Parte I: Hábitos de compra y consumo

1. De un total del 100%, ¿qué porcentaje le asignaría usted a **cada uno** de los siguientes tipos de aceite que suele consumir habitualmente en su hogar?

Aceite de oliva	
Aceite de maravilla (vegetal)	
Aceite de colza	
Aceite de maíz	
Aceite de pepita de uva	
Otro tipo de aceite ¿cuál?	

2. En una escala de 1 a 7 donde 1 significa que "no es importante" y 7 que "es muy importante", podría valorar **cada una** de las siguientes acciones que usted considera que se deberían realizar en el mercado local para estimular el consumo habitual de aceite de oliva.

Si no consume aceite de oliva pasar a 3
Si consume aceite de oliva pasar a 4

Mayor promoción de aceite de oliva en los medios de comunicación	
Mayor promoción de aceite de oliva en los puntos de venta	
Informando en los medios de comunicación los beneficios para la salud de este producto	
Destacar en el envase del producto los beneficios de una dieta mediterránea	
Promocionando en los puntos de venta el aceite de oliva elaborado en Chile	
Realizar promociones con otro tipo de productos, como por ejemplo vegetales procesados	
Me da lo mismo, de igual forma no voy a consumir este tipo de aceite	

3. En una escala de 1 a 7 donde 1 significa que "no es importante" y 7 que "es muy importante", podría valorar **cada una** de las siguientes razones por las cuales **no suele consumir** aceite de oliva habitualmente.

Pasar a la pregunta 16

No me gusta su sabor	
Tiene un precio muy elevado en relación a otros aceites	
No estoy acostumbrado a consumir aceite de oliva	
Es un producto que no he incorporado a mis hábitos de alimentación	
La disponibilidad de este producto es reducida	
Nunca me ha llamado la atención consumir este producto	
Las campañas publicitarias de aceite de oliva no son masivas	
A mi familia no le gusta este tipo de aceite	
Otra razón ¿Cuál?	

4. En una escala de 1 a 7 donde 1 significa que "no es importante" y 7 que "es muy importante", podría valorar **cada una** de las razones de por qué **consume** aceite de oliva.

Es un aceite que beneficia la salud	
Tengo un mayor poder adquisitivo que antes	
Es un producto bajo en calorías	
Es un aceite más natural que otros	
Me gusta su sabor y olor	
Por recomendación médica	
He decidido incorporar productos más saludables a mi dieta de alimentación	
Otra razón ¿Cuál?	

5. ¿Con qué frecuencia en promedio suele comprar aceite de oliva? **(Respuesta única).**

1 vez por semana	1
1 vez cada 15 días	2
1 vez al mes	3
1 vez cada 2 meses	4
1 vez cada 3 meses	5
2 veces al año	6
1 vez al año	7
Otra ¿Cuál?	8

6. ¿Qué volumen suele comprar usualmente en cada ocasión? **(Respuesta única).**

100 ml	1
250 ml	2
500 ml	3
750 ml	4
1.000 ml	5
Otra ¿Cuál?	8

7. ¿Cuánto suele gastar en promedio cada vez que compra aceite de oliva?_______________

8. De un total del 100%, ¿qué porcentaje le asignaría usted a cada uno de los siguientes lugares en donde suele comprar aceite de oliva?

Supermercados (como jumbo, unimarc, líder, entre otros)	
Tiendas mayoristas	
Tiendas gourmet	
Tiendas del retail (como falabella, parís, ripley)	
Directamente al productor	
Ferias artesanales de la ciudad	
Otras tiendas ¿Cuáles?	

9. ¿En qué ocasiones suele consumir aceite de oliva? **(Respuesta múltiple).**

Cuando celebro hechos importantes de la familia (Cumpleaños, aniversarios, logros familiares)	1
Cuando invito a comer a mi casa a mis amigos o colegas	2
Cuando voy a un restaurant con la familia o con amigos	3
Fin de semanas con la familia	4
En fiestas religiosas (semana santa, navidad)	5
No hay una ocasión fija	6
Otra ocasión ¿Cuál?	7

UNIVERSIDAD DEL BÍO-BÍO

Comité Asesor de Bioética y Bioseguridad
DIRECCIÓN DE INVESTIGACIÓN
Universidad del Bío - Bío

10. Indique los usos que suele darle al aceite de oliva en **cada una** de las ocasiones que se presentan a continuación, y si estos son habituales u ocasionales.

	**Habitual**	**Ocasional**
En todo tipo de ensaladas		
En ensaladas especiales (mediterráneas)		
Para freír		
Para cocinar comidas especiales		
Para sazonar		
En todo tipo de comidas		
Para preparar entradas		
Otra ¿Cuál?		

11. ¿Cómo se informó de la disponibilidad que existe actualmente de aceite de oliva en el mercado? **(Respuesta múltiple).**

Por la publicidad de la televisión	1
Por medio de la radio	2
Por el periódico	3
Por la publicidad en el supermercado	4
Por un amigo / familiar	5
Otra ¿Cuál?	6

12. De un total del 100% de las compras de aceite de oliva que usted realiza ¿qué porcentaje le asigna a los siguientes países de procedencia al aceite de oliva que suele consumir?

Chile	
España	
Italia	
Argentina	
Grecia	
Otro ¿Cuál?	

13. En una escala de 1 a 7 donde 1 significa que "no es importante" y 7 que "es muy importante", podría valorar **cada una** de la importancia que usted le da a los siguientes atributos a la hora de comprar aceite de oliva.

**Atributo**	**Puntuación**
Envase	
País de procedencia	
Volumen (ml)	
Precio	

**Atributo**	**Puntuación**
Color	
Olor	
Sabor	
Textura	
Apariencia	

14. Centrándonos ahora en un aceite de oliva de su total preferencia, le pido que ordene los siguientes atributos preferidos por usted de mayor a menor, **siendo el número 1 el más preferido y 3 el menos preferido.**

	Plástico	Vidrio	Lata	Otro
**Envase**				
	Chile	España	Italia	Otro
**Procedencia**				
	250	500	1000	Otro
**Volumen (ml)**				
	Amarillo	Verde		
**Color**				
	Frutoso	Amargo	Picante	
**Sabor**				
	Opaco	Claro		
**Apariencia**				

15. ¿Qué características tenía el aceite de oliva que compró por última vez y cuánto pagó por él? **Sólo marcar una alternativa por cada una de las características.**

**Característica**	Plástico	Vidrio	Lata	Otro
**Envase**				
	Chile	España	Italia	Otro
**Procedencia**				
	250	500	1000	Otro
**Volumen (ml)**				
	Amarillo	Verde		
**Color**				
	Frutoso	Amargo	Picante	
**Sabor**				
	Opaco	Claro		
**Apariencia**				

$ ______________________________

Parte II: Aspectos Psicográficos

En esta sección debe responder una serie de preguntas que tienen como objetivo estudiar algunos aspectos Psicográfico de los encuestados.

16. **(Escala de valores)** En una escala de 1 a 7 donde 1 significa que "no es importante" y 7 que "es muy importante", ¿qué importancia tiene para usted cada uno de los valores que se presentan a continuación como principios que orientan su vida personal?

	**Ítem**	**Nota**
1	Autorrealización	
2	Autoestima	
3	Entusiasmo	
4	Placer y disfrute de la vida	
5	Relaciones de afecto con los demás	
6	Seguridad	
7	Sentido de pertenencia a un grupo	
8	Sentido de cumplimiento de objetivos	
9	Ser respetado	

Comité Asesor de Bioética y Bioseguridad
DIRECCIÓN DE INVESTIGACIÓN
Universidad del Bío - Bío

17. **(Fobia a los alimentos)** En una escala de 1 a 7 donde 1 significa que **"está totalmente en desacuerdo"** y 7 que **"está totalmente de acuerdo"**, le pedimos que valore **cada uno** de los ítems que se presentan a continuación relacionados con la fobia a los alimentos.

	Ítem	Nota
1	Constantemente pruebo comidas o alimentos nuevos o diferentes	
2	No confío en comidas o alimentos nuevos	
3	Si no sé lo que hay en una comida o alimento, no lo probaré	
4	Me gustan las comidas o alimentos de países diferentes	
5	La comida étnica parece demasiado extraña para comer	
6	En ocasiones especiales, probaría comidas o alimentos nuevos	
7	Me da miedo comer cosas que nunca he probado antes	
8	Soy muy particular respecto a las comidas que como	
9	Yo comería casi cualquier cosa	
10	Me gusta probar restaurantes étnicos nuevos	

18. ¿Cómo considera que es su estado de salud actual? **(Respuesta única).**

Bueno	1
Regular	2
Malo	3

19. ¿Con qué frecuencia suele practicar deportes? **(Respuesta única).**

No práctico deporte	1
Diariamente	2
Tres a cuatro veces por semana	3
Dos veces por semana	4
Una vez por semana	5
Una o dos veces al mes	6

20. Su consumo diario de alimentos, ¿suele ser sano y saludable? **(Respuesta única).**

Siempre	1
A veces	2
Nunca	3

Parte III: Aspectos socio-demográficos

Por último, y ya para finalizar esta encuesta, podría decirme:

21. Sexo

	Masculino		Femenino

22. Cuál es su año de nacimiento ________

23. Incluyéndose usted, ¿cuántas personas de su familia viven en su hogar? ______________

24. Podría decirme las edades de todos los miembros de su familia que viven en su hogar, comenzando por la suya.

25. Sería tan amable de decirme ¿cuál es el presupuesto aproximado mensual **que su familia** destina para hacer las compras de alimentos? $______________

26. ¿Cuál es su estado civil?

Casado (a) **(No responder pregunta 30)**	1
Soltero (a)	2
Separado (a)	3
Divorciado (a)	4
Viudo (a)	5

27. ¿Tiene hijos? (*Los que no consumen aceite de oliva responder las preguntas 28, 30 a la 32, 34 y de la 36 a la 41*).

No	Sí	Cuántos:

28. ¿Ha vivido alguna vez en otro país?

No **(Pasar a la pregunta 30)**	Sí	¿En cuál?
		¿Cuánto tiempo?

29. ¿Cree usted que su experiencia de haber vivido en el extranjero, influenció en su consumo de aceite de oliva?

No	Sí	¿Por qué?

30. **(Los casados no responder)** ¿Actualmente tiene pareja?

No **(Pasar a la pregunta 34)**	Sí

31. ¿De qué nacionalidad es su esposo (a) / pareja?
____

32. ¿Su esposo (a) / pareja actual ha vivido en otro país?

No **(Pasar a la pregunta 34)**	Sí	¿En cuál?
		¿Cuánto tiempo?

33. ¿Cree usted que la experiencia de su esposo (a) / pareja actual haber vivido en el extranjero, influenció en su consumo de aceite de oliva?

No	Sí	¿Por qué?

34. ¿Alguna persona cercana a usted ha vivido en otro país?

No **(Pasar a la pregunta 36)**	Sí	¿Qué parentesco tiene con esa persona?
		¿En qué país vivió?
		¿Cuánto tiempo?
		¿Nacionalidad?

UNIVERSIDAD DEL BÍO-BÍO

Comité Asesor de Bioética y Bioseguridad
DIRECCIÓN DE INVESTIGACIÓN
Universidad del Bío - Bío

35. ¿Cree usted que la experiencia de esa persona cercana de haber vivido en el extranjero, influenció en su consumo de aceite de oliva?

No	Sí	¿Por qué?

36. ¿Cuál es el rol que usted desempeña en su hogar?

Vivo solo	1
Cabeza de familia	2
Cónyuge	3
Ama de casa	4
Hijo (a)	5
Abuelo (a)	6
Otro (a)	7

37. ¿Podría indicarme en qué sector de la ciudad de Chillán vive?

____

38. ¿Cuál es su nacionalidad? ________________

39. Sería tan amable de indicarme su nivel de estudios **finalizados.**

Educación básica	1
Educación media	2
Centro de formación técnica	3
Educación técnico profesional	4
Universitaria	5
Postgrado (diplomado, magister, doctorado)	6

40. ¿Me podría indicar aproximadamente en qué rango de ingresos mensuales promedio se encuentra su familia?

Menos de $300.000	1
Entre $300.001 y $600.000	2
Entre $600.001 y $900.000	3
Entre $900.001 y $1.500.000	4
Entre $1.500.001 y $2.500.000	5
Entre $2.500.001 y $3.500.000	6
Más de $3.500.000	7

41. ¿Cuál es su situación laboral actual?

**Inactivo (sin actividad)**	
Estudiante	1
Ama de casa	2
Desempleado	3

**Trabajadores independientes (rama de actividad)**	
Agricultura, pesca y caza	4
Minas y canteras	5
Industrias manufactureras	6
Electricidad, gas y agua	7
Construcción	8
Comercio	9
Transportes, almacenamiento y comunicaciones	10
Bancos y financieras	11
Servicios comunales, sociales y personales	12

**Trabajadores dependientes (grupos de ocupación)**	
Profesionales técnicos y afines	13
Gerentes, administradores y directivos	14
Empleados de oficina	15
Vendedores y afines	16
Funcionarios públicos	17
Funcionarios de las fuerzas de orden y seguridad	18
Agricultura, ganadería, pesca y caza	19
Conductor de transportes	20
Artesanos y operarios	21
Obreros y jornaleros	22
Trabajador en servicios personales	23
Trabajadores en ocupaciones no identificadas	24

Parte IV: Choice

	Perfil	Selección
**Bloque:**	P1	
	P2	
	P3	
	P4	
	P5	
	P6	
	P7	
	P8	
	P9	

Orden de preferencia de la degustación

Elección 1	Elección 2	Elección 3

Le agradecemos su tiempo y el interés que ha tenido para contestar esta encuesta. Espero que las preguntas no le hayan generado ningún tipo de inconveniente. La información que nos ha proporcionado es muy valiosa para nosotros y nos permitirá obtener conclusiones muy importantes. Muchas gracias.
