# Supplementary material for: Heterogeneity and nonlinearity in consumers’ preferences: An application to the olive oil shopping behavior in Chile
Source: PLoS One. 2017 Sep 11;12(9):e0184585. doi: 10.1371/journal.pone.0184585 (PMC5593193; doi:10.1371/journal.pone.0184585)
Supplement: S2 File — (DOCX) [file pone.0184585.s002.docx]

| **Universe** | Persons over 18 years belonging to census districts (cd): cd1, cd2, cd3, cd13, cd14, and cd15 in the city of Chillán. |
| --- | --- |
| **Sample size** | 221 surveys. |
| **Error** | 6.72% (P = 0.5) |
| **Significance level** | 95.5% (Z = 2) |
| **Sampling** | Random sampling stratified by sex, age, and geographic zone. |
| **Pre-test and pilot**  **questionnaire** | 30 and 80 questionnaires, respectively. |
| **Field work** | Downtown at the mall and different supermarkets. |
| **Study date** | January to March 2012 |
